# The California Border Health Collaborative: A Strategy for Leading the Border to Better Health

**DOI:** 10.3389/fpubh.2015.00141

**Published:** 2015-05-26

**Authors:** Charles Edwards Matthews, Wilma Wooten, María Gudelia Rangel Gomez, Justine Kozo, April Fernandez, Victoria D. Ojeda

**Affiliations:** ^1^Health and Human Services Agency, County of San Diego, San Diego, CA, USA; ^2^Joint Doctoral Program, Global Health, San Diego State/University of California San Diego, San Diego, CA, USA; ^3^Public Health, Health and Human Services Agency, County of San Diego, San Diego, CA, USA; ^4^U.S.-Mexico Border Health Commission, Mexico Section-Secretary of Health, Tijuana, Mexico; ^5^California Department of Public Health, Office of Binational Border Health, San Diego, CA, USA; ^6^School of Medicine, University of California San Diego, San Diego, CA, USA

**Keywords:** border health, collaboration, live well San Diego, border collaborative, U.S.–Mexico border, U.S.–Mexico border health commission, border collective impact

## Abstract

There are hundreds of people and organizations working on border health issues in the California–Baja California border region trying to protect and improve health. These efforts are being conducted without a collaborative structure that integrates jurisdictions and organizations. Thus, there is a need to coordinate these organizations to work together and benefit from their collective effort and each other’s best practices. The outcome of such an effort could effectively improve the health in the border region. The newly developed “*California Border Health Collaborative*” unites organizations and provides the leadership and collaborative culture to positively improve the health of the border region; it is referred to as the “Collaborative.” This article describes the developmental process of this Collaborative, including partner engagement, governance, strategic planning, key elements for success, the roles of multi-level jurisdictions, and policy implications. This paper focuses on describing the preparation and processes that created the U.S./California side of this binational collaborative effort and is a strong reflection of the theory of border collaboration as described by Denman and De Sonora (1) in “Working beyond Borders: A Handbook for Transborder Projects in Health.”

## Background

### Defining the border region

The California–Baja California border includes two counties on the U.S. side, San Diego and Imperial and spans approximately 322 km along the Baja California border.

Imperial County has a total area of 4,482 mi^2^ or about twice the size in total square miles as the State of Delaware. Imperial County is located in the Imperial Valley, in the far southeast of the U.S. State of California, bordering Arizona and Mexico (http://www.co.imperial.ca.us/index.asp). In contrast, San Diego County is large and diverse and serves as a microcosm of the country. With a population of 3.2 million, San Diego is the fifth largest county in the United States. The San Diego and Tijuana border region is home to the busiest land border crossing in the world, the San Ysidro border crossing. There are six ports of entry on the California–Baja California border with 63,048,683 northbound border crossings in 2010, with San Ysidro having 44,009,770 (70%) of these total northbound crossings in 2010.

In addition to the nearly 400,000 San Diego County residents of Hispanic origin, people crossing the California–Baja California border are a major part of the San Diego County economy and culture. This is a daily, highly mobile population that traverses two distinctly different countries. Due to these and other variables, the border region has significant health challenges and a great need for a collaborative approach to health.

According to the California Department of Public Health (CDPH), Office of Binational Border Health (OBBH) *Border Health Status Report* to the Legislature, 2011, 2012 and others; tuberculosis (TB), obesity, and diabetes are significant health issues affecting the border region (2–5).

### Precursors to and key players in the collaborative

From 2009 to 2010, there was a convergence of factors among several San Diego organizations that perform border health work, which were precursors to the development of the California Border Health Collaborative. These events and key organizations are outlined below and contextualize the Collaborative’s formation and evolution.

#### County of San Diego Health and Human Services Agency

In July 2010, The San Diego County Board of Supervisors’ commitment to regional wellness across jurisdictions, systems, and populations led to the development of *Live Well San Diego*, a long-term vision to support healthy, safe, and thriving communities throughout San Diego. The foundation of *Live Well San Diego* is based on the concept of collective impact – the idea that health and well-being for all residents could be achieved more effectively by establishing collaborative partnerships rather than individual organizations working alone.

#### California Department of Public Health, Office of Binational Border Health

The CDPH, OBBH worked closely with HHSA as both a conduit and partner to federal and local health departments. With a mission to facilitate communication, coordination, and collaboration among California and Mexico health officials, health professionals, and communities in order to optimize border and binational health, their expertise in border health issues and ability to convene partners was instrumental in the development of the Collaborative and its inception.

#### Local Academia

Both San Diego State University (SDSU) and the University of California San Diego (UCSD) host numerous well-established research teams that examine border issues. These academics were well-versed in doing cross border research and had success in building binational, collaborative partnerships needed to carry out their projects. These successes were increasingly intersecting with the County of San Diego HHSA, OBBH, and local non-profit organizations. Examples include UCSD’s TB prevention research exploring the use of video direct observation therapy (VDOT) in the border region, a project in collaboration with County HHSA TB Control, HIV, and STD surveillance and treatment and cross border research that utilized the County HHSA’s Public Health Laboratory for specimen testing. It was deemed that academia would be an important partner in the Collaborative.

#### U.S.–Mexico Border Health Commission

In parallel to the examples listed above, the U.S.–Mexico Border Health Commission (USMBHC) had been supporting the San Diego County Public Health Department, as well as OBBH on specific health issues such as TB, communicable disease surveillance as well as combating chronic diseases in the region. The USMBHC provided technical assistance, conferences, trainings, and regional planning support to state and local government branches.

In 2010, the USMBHC sponsored what would prove to be a turning point for border collaboration in California, the *Leaders Across Borders (LAB)* program. *LAB* is a yearly, 10-month long program that teaches and mentors health professionals and community leaders to design and implement projects to address the needs of underserved communities in the U.S.–Mexico Border region. Participants learn how to effectively collaborate with one another by developing skills in health diplomacy and gaining a deeper understanding of cultural differences and binational health care systems.

Several leaders from the County of San Diego, OBBH, local Universities, and local non-profit organizations were participants and/or facilitators in the inaugural LAB program. Through the training and mentorship of the USMBHC program, coupled with the County’s *Live Well San Diego* framework, OBBH’s integration approach and local academia expertise, a collaborative paradigm of border health leadership was evolving. Furthermore, the California border region’s leaders were adhering to what researchers throughout the entire U.S.–Mexico border had been finding: due to the health challenges in our border region, adequately addressing the needs of a border community requires effective transnational communication, beginning at the local level and then involving regional/state and federal governments (1).

### Texas provides an example

Up until this point, the above San Diego and California border health actors and their organizations were working on various health issues, divided by topic, jurisdiction, or specific interest (e.g., research vs. service provision). In June 2010, many of these same border health organizations were in Washington, D.C., for an annual Border Health conference and participants attended a reception sponsored by the Texas Medical Association. The dual purpose of this conference was to share expertise on relevant border issues in which all the U.S. Border States participated and Texas specifically utilized it as an opportunity to educate their national legislators and the U.S. Congress on border health issues. Texas was extremely organized and effective in accomplishing these goals. They presented a structured, united front, having their representatives and senators engaged and working for the betterment of the health of their border region. The San Diego County HHSA, OBBH, Project Concern International (PCI), and UCSD participants took note and, in witnessing this example, reflected on what was possible in the border region of California.

## Approach/Methods

According to *Working Beyond Borders*, building an effective binational approach and/or organization takes the right people, the right environment, and the right organizational structure (1). In San Diego, with the right people and environment taking shape, in order to develop the right organizational structure, ensuring trust was an essential step toward achieving success. To continue building trust among present and future border partners, the following were necessary: collaborative leadership at a high level, transparency, USMBHC support, and an initial team-building project. Additionally, strategic alignment and a common vision for border health were important in order to move forward.

Between June 2010 and January 2011, County of San Diego HHSA and OBBH started to discuss the possibility of trying to develop a collaborative that would have the ability to unite the experts and champions working in the California border region and eventually engage our partners in Baja California, Mexico. Early discussions identified possible ideas/benefits of a collaborative group:

1. Increase understanding and knowledge of the different programs working in the local border region.
2. Increase ability to collaborate/partner toward common goals and priorities.
3. Combine efforts to attract and apply for financial resources for our border region.
4. Streamline efforts, while building partnerships that would produce powerful and effective results in addressing public health issues.
5. Increase efficiency in our regional response to public health emergencies or urgent issues.
6. Integrate Imperial County, San Diego County, OBBH, and USMBHC in strategic planning efforts for our border region.

Convening this group would build on the Border Childhood Obesity Forum that took place in August 2010, which emerged as an outcome of a LAB project and successfully brought together organizations and people working on the obesity issue in the California–Baja California border region. In addition, it was agreed that if successful, once the California side was working effectively in collaboration, the group would then approach and engage counterparts in Baja California. The Public Health Officer for the County of San Diego and the Chief of OBBH were the conveners/facilitators of this new collaborative with both organizations’ staffs working to coordinate its presence in the community.

### Selection of Target Partners

The San Diego County HHSA and OBBH completed an informal stakeholder analysis and identified individuals and organizations that needed to be at the table. A broad cross-section of stakeholders from various levels of government (e.g., Federal, State, Local) and from diverse organizations (e.g., academic, non-profit, coalitions, advocacy groups) were invited to the first meeting to discuss the possibility of forming a local border health collaborative.

Along with these foundational or “convening” governmental stakeholders, other organizations brought powerful expertise to the collaborative. Many researchers from UCSD and SDSU had been performing significant border health research for years and have built effective working relationships with organizations on both sides of the border. The same can be said of non-government organizations or non-governmental organizations (NGOs), like PCI, San Ysidro Health Center, and others with direct service programming on both sides of the border and/or in the shared border region. Their leadership and link with academia was a key to successful border health work in the region. It was felt that this collaborative could provide an excellent avenue and opportunity to bridge research and operations in government and NGOs to affect the health of the region. Reiterating that effectively impacting the health of the region cannot be done by government alone, other organizations are needed to share their expertise in order to achieve the collective impact that this collaborative hopes to achieve (6).

### Collaborative Stakeholder Survey

Following the first meeting, a survey was emailed to all the attendees. The survey aimed to obtain information, identify, and share a list of all entities involved in border health work in the California border region and secondly, to obtain participants’ opinions regarding involvement in the group (e.g., how to structure the group, how often to meet, how best to communicate), and finally, whether they would be interested in participating in a trip in June 2011 to Washington, D.C., as part of the California Caucus representing the region and the Collaborative at the Border Conference, organized by the Texas Medical Association.

## Results

Over 20 organizations and 40 attendees were identified and participated in the Collaborative’s first meeting on Feb 17, 2011. There was representation from all three organizational sectors of government, non-profit, and academia. The number of border actors currently on the list of participants has swelled to over 183. At the conclusion of this first meeting, participants agreed by consensus to move forward and develop the collaboration, have the County Public Health Officer and Chief of OBBH lead the meeting, and lastly to send out a survey with key questions about how to move forward.

### Stakeholder Survey Results

Results to the survey demonstrated overwhelming support to move forward with developing the Border Health Collaborative. There were 25 respondents to the survey and 100% of those surveyed voted to participate in the Collaborative going forward, over half of the respondents wanted the group to be formally governed as opposed to an informal networking group only, 85% wanted to meet quarterly or bi-monthly, 85% wanted to meet in person with the ability to call in if needed, and 60% would plan to or might attend the Border Health Conference in June in Washington, D.C.

#### Initial Project Identified by the Survey

During discussions and per the survey, participants agreed to prepare for the upcoming Border Health Conference in Washington, D.C. in June (4 months away). Approximately, 10 conference participants represented government, academia, and non-profit. Preparation for the conference involved gathering information about our California border region and our newly formed Collaborative and sharing it during the conference. While the Collaborative consisted of many different organizations attending from our region, the group would attempt to speak in one voice. This translated to developing a one page document that presented vital information about the newly formed collaborative and health information about the region (see Supplementary Material on *California Border Health Collaborative Information Sheet)*.

### Collaborative Evolving

The Collaborative decided to meet monthly for the first year. Each meeting had a defined theme and guest speaker, based on the interests of Collaborative members. After the first year, meetings were taking place every 2 months. After an “initial project” of preparing a Collaborative information sheet for a border conference in Washington, D.C. was completed in June 2011, the Collaborative next produced a Mission, Vision, and Strategic Goals (7). This California Border Health Collaborative Strategic Plan was based on and completed after reviewing and considering the World Health Organization Millennium Goals, Centers for Disease Control and Prevention (CDC) Healthy People 2020, USMBHC Healthy Border 2020, OBBH Strategic Goals as well as the HHSA County of San Diego *Live Well San Diego* Framework. The Collaborative ensured that all strategic plans from all levels of government were considered. This commitment to strategic alignment was also reflected when the Collaborative members developed a strategic plan to include a vision, mission, and strategic objectives (See Supplementary Material on California Border Health Collaborative Strategic Plan). Upon finishing the Collaborative Strategic Plan, on December 16, 2011, the team developed four sub-committees that reflected the priorities of the collaborative:

- *The Steering Committee* – goals include developing agendas, strategic direction, alignment, and decision-making authority.
- *The Communications Committee* – goals include facilitating communication, through the development of a bilingual and binational web platform that is a hub for sharing resources.
- *The Resource Development Committee* – goals include promoting funding collaboration between public, private, and academic institutions that are submitting grant proposals focused on border health issues.
- *The Binational Engagement Committee* – goals include seeking appropriate and effective methods for engaging our Baja California partners to create an efficient and mutually beneficial binational collaborative.

By April 2012, just over a year after initially forming, the California Border Health Collaborative began developing a strategy for engaging Baja California to form a local *binational consortium*.

Until this point, the California Border Health Collaborative had built a strong, cohesive collaborative on the U.S. side of the border. It was effectively sharing and leveraging local expertise to address many border health issues. Even more importantly, the Collaborative had reached a key stage in which it was ready to effectively engage its Baja California, Mexico counterparts in a manner that would prove extremely powerful (Table 1). Discussion of the process of engaging the Baja California partners is outside the scope of this paper.

**Table 1:** **Timeline – California border health collaborative process and results**.

11/02/10	Generated action items at the HHSA Quarterly Border Health meeting which evolved into the Border Health Collaborative (now Border Health Consortium of the California)
2/17/11	First meeting of local border organizations, regional universities and colleges, community agencies, and government
6/11	10 Participants attended the border conference in Washington, D.C.
8/18/11	Began strategic planning
10/20/11	Completed strategic plan
12/16/11	Formed four sub-committees
4/19/12	Binational engagement committee developed strategy to engage Baja California; first draft of charter reviewed
6/26/12	Overview of final strategic plan
10/12/12	The first binational engagement meeting with Mexico occurred in Baja

## Discussion

### Building Trust

In subsequent meetings, there was an overarching focus on building trust between each other and in the process of developing the collaborative. There were four key contributing factors that helped build trust in the group: transparency, collaborative leadership, USMBHC support, and an initial project. These are outlined below.

#### Transparency

In striving for transparency, there was a conscious decision made that all aspects of developing this collaborative needed to be discussed with the group in open meetings. The group agreed and decided the structure of the collaborative to include wanting to have the two local government agencies (San Diego County Public Health Department and OBBH) leading the meetings. In addition, all members of the group wanted to share in the coordination of the Collaborative. This meant the forming of a leadership steering committee with participation open to all members.

#### Collaborative Leadership

According to Madeleine Carter, from the Center for Effective Public Policy, one of the key qualities of a collaborative leader is the ability to share knowledge, power, and credit (8). As noted above, key, convening, government organizations were leading in this way and brought these leadership characteristics to the Collaborative and the process of developing it. In addition, there was great optimism by the leaders in addressing the health needs of the border region. The leadership and members of the Collaborative understood that in order to advance as a region, it would be essential to foster opportunities for authentic cross border communication and collaboration. As defined in *Working Beyond Borders*, true collaboration involves the sharing of power, responsibilities, decision-making, and accountability, in order to achieve positive outcomes defined by mutual goals (1).

#### USMBHC Support

Up until this point, the USMBHC had been instrumental in at least three ways in the formation of the Collaborative. The USMBHC has supported the training of leaders via the LAB program. This Collaborative is the type of project or initiative that the LAB program challenged its graduates and affiliates to undertake. Secondly, during this time, the local San Diego border health community, as well as the rest of the border had a champion collaborative leader. This leader was the General Manager of the U.S. Section of the USMBHC, the late Dan Reyna; he had forged effective working relationships in the San Diego region with OBBH, the County of San Diego and others and had mentored and supported key staff in a way that fostered collaboration. In all of his interactions with local border health staff, he espoused a core belief that the power and impact of border health work occurs at the local level. He did this in a way that empowered and inspired staff and their local leaders. He is often remembered by local border staff as saying, “…this is your sandbox, you’re in charge…tell me how I can help.”

Dan provided an ongoing operational example that epitomized the role of the USMBHC, …the roles of the Commission include: institutionalize a domestic focus on border health which can transcend political changes, become a venue of broad participation by health professional and others interested in improving border health, promote social and community participation, be a catalyst, be a policy advocate, increase resources for the border, encourage self-responsibility for health (9).

#### Initial Project

By developing the *California Border Health Collaborative Information Sheet* (see Supplementary Material) to share in Washington, D.C., the members began identifying priorities and visualizing themselves as one unified Collaborative. The team members also had an appreciation for the expertise and information that the San Diego/Imperial area held. The process itself was effective in helping the Collaborative team members connect and build trust.

### Policy Implications

Reflecting on the ongoing Collaborative building process, there are at least three policy recommendations that can be derived. These include institutionalizing the Collaborative in government agencies, ensuring a commitment to training border leaders via programs like *LAB* and ensuring governments consider each other’s strategic goals and objectives when developing their own strategic plans.

- Ensuring this Border Health Collaborative continues to be a recognized piece of the working infrastructure of the County and OBBH. This is accomplished by solidifying the commitment from the top leadership that this Collaborative serves as an integral tool to approaching border health. This has been done in San Diego with the Director of HHSA, the Public Health Officer, the Chief of OBBH, as well as the California Public Health Officer, all demonstrating commitment to this Collaborative. This Collaborative has already adjusted to leadership replacement and continued just as strong when the Chief of OBBH retired and his successor did not miss a step in continuing their commitment. This robust infrastructure also continued when the County of San Diego, Public Health Chief of Operations left and moved to another position in HHSA. Ensuring institutionalization of the group is vital.
- Commitment to participating in *LAB* on a yearly basis with new leader participants and facilitators is a key to the success of the Border Health Collaborative and enhances our ability to be effective. The *LAB* assists the collaborative in building capacity and training health professionals to be effective leaders in the border region. The Border Collaborative leadership encourages its members and their organizations to send staff to participate in the program. These graduates then in turn serve as succession potential in their own organizations as well as for the Border Health Collaborative. This all serves as building the institutionalization of the Collaborative. Also, the *LAB* requires participants to work on a binational health project. The Collaborative could participate in the project selection process by offering suggestions or ideas to the *LAB* participants that help support the local border health landscape. These project possibilities can be strategically developed from the Collaborative’s strategic plan.
- Lastly, requiring government organizations to consider each other’s strategic planning when developing their own is key. When the county and state health departments develop their strategic goals, they should review Federal CDC and USMBHC goals and objectives. In turn, the federal government should be seeking input of state and local border stakeholders The USMBHC has recently done this by using the advisory board of the State Office of Border Binational Health and seeking their input in strategic and priority planning. This should be imbedded in the process of strategy development. With more strategic alignment comes the ability to leverage resources and a more profound impact on the regions health (10).

## Conclusion

As discussed throughout this paper and according to previous work by Denman and her colleagues, collaboration among U.S. border partners to impact the health of the border region can be achieved (1, 11, 12). This can be done by forming a Collaborative that seeks to harness the expertise of various levels of government, academia, and NGOs in local border regions. In order to be successful, transparency, trust, collaborative leadership, and support from the USMBHC are essential components. To ensure sustainability, supportive policies should be considered, such as, institutionalizing the Collaborative within government agencies, demonstrating commitment to training border leaders and lastly, aligning government organizations strategic goals and objectives. When all of this occurs, then the local, U.S. side of the border is effectively aligned to engage the local Mexico side of the border. The Collaborative is now ready to expand and become binational in its health efforts. This current binational engagement process will be described in a future article.

## Conflict of Interest Statement

The authors declare that the research was conducted in the absence of any commercial or financial relationships that could be construed as a potential conflict of interest.

## Supplementary Material

The Supplementary Material for this article can be found online at http://journal.frontiersin.org/article/10.3389/fpubh.2015.00141
