# Supplementary material for: The California Border Health Collaborative: A Strategy for Leading the Border to Better Health
Source: Front Public Health. 2015 May 26;3:141. doi: 10.3389/fpubh.2015.00141 (PMC4443723; doi:10.3389/fpubh.2015.00141)
Supplement: Supplementary file 1 [file Data_Sheet_1.PDF]

Supplementary Material:

The California Border Health Collaborative: A Strategy for Leading the Border to Better Health

Appendix 1. California Border Health Collaborative Information Page (s)

California Border Health Collaborative

Collaborative Members

California Department of Public Health: Emergency Preparedness Office; Office of Binational Border Health (COBBH): COBBH Core Program, COBBH Early Warning Infectious Disease Surveillance Program, COBBH U.S.- Mexico Border Health Commission-California Outreach Office, STD Control Branch
California State University, San Marcos (CSUSM): School of Anthropology, National Latino Research Center
Centers for Disease Control and Prevention (CDC)
Clinicas de Salud del Pueblo
Council of Community Clinics
Grossmont-Cuyamaca Community College District
Health Initiative of the Americas, University of California, Berkeley
Imperial County Public Health Department
International Community Foundation
North County Health Services
Planned Parenthood of the Pacific Southwest
Project Concern International: California Border Healthy Start, Proyecto Salud TB
San Diego City College
San Diego County Health and Human Services Agency: Border Health Program; Central and South Regions; STD, HIV, and Hepatitis Branch; TB and Refugee Health Branch
San Diego Medical Society
San Diego State University: Graduate School of Public Health, Institute for Public Health
San Ysidro Health Center
Sekure Healthcare
University of California, San Diego: School of Medicine, Division of Global Public Health, HIV Binational Research Studies

Website

http://www.cdph.ca.gov/programs/cobbh/Pages/CaliforniaBorderHealthCollaborative.aspx

June 2011

Background

In February 2011, the California Office of Binational Border Health (COBBH); County of San Diego, Health and Human Services Agency (HHSA); and Imperial County Public Health Department (ICPHD) formed an alliance to create the California Border Health Collaborative.

Mission

"To enhance communication, coordination, and collaboration among public, private, and academic institutions in order to protect and improve the health of individuals and communities along the California-Baja California Border Region."

California's Unique Border Region: San Diego and Imperial Counties

- *High Cross-Border Mobility*
  - In 2010, a combined daily northbound average of 72,482 vehicles and 40,384 pedestrians passed northbound through the region's six border crossings
- *Dynamic Economy*
  - Agriculture: With a combined total of more than 7,100 farms, the region leads the nation in avocado, flower, citrus, hay and forage production
  - Small Business: Employing more than half of the region's workforce, businesses with less than 500 employees comprise 97% of San Diego and Imperial County firms
- *Culturally and Demographically Diverse Region*
  - Over 80% of Imperial County residents are Hispanic/Latino
  - San Diego County has more Indian Reservations than any other county in the United States
  - With more than 4,000 new arrivals in 2010, San Diego County is the nation's number one refugee resettlement community
  - In 2009, a total of 113,755 military personnel (57,060 Marines and 56,695 Navy) lived in San Diego County

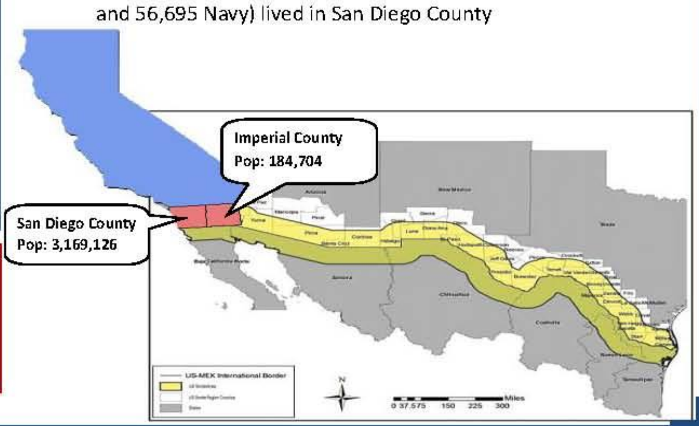

Appendix 1 cont.

California Border Health Collaborative

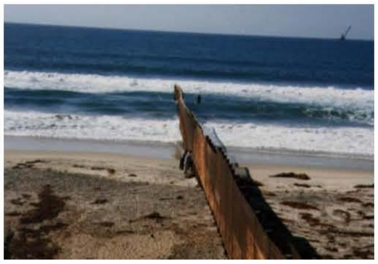

Border Fence Extending to Pacific Ocean

California Border Region Statistics

Border Crossings

- There are six ports of entry with 63,048,683 northbound border crossings in 2010; San Ysidro is the **busiest land Port of Entry in the world** with 44,009,770 northbound crossings in 2010.

Demographics

- In 2009 the total estimated population of the two California border counties was 3,353,830 (184,704 in Imperial County and 3,169,126 in San Diego County), representing 10.5 percent of California's population.

- From 2000 to 2009, the border region experienced steady population growth. Imperial County's population increased by 28.5 percent, more than double the rate of increase in San Diego County (11.7%) and in California overall (13.4%) during the same period.

Public Health Issues

Infectious Diseases

Tuberculosis:

- In 2009, Imperial County had the highest rate of TB (18.9 per 100,000; 39 cases) in California
- In 2009 San Diego County had the 9th highest rate of TB in California (7.0 per 100,000; 223 cases)

HIV/AIDS:

- Between April 2006 and September 2010, there were 4,428 case reports of HIV in San Diego County and 56 case reports in Imperial County
- San Diego County has had 14,512 AIDS cases and Imperial County has had 234 AIDS cases since 1983

Chronic Diseases

Obesity:

- In 2009, the majority of the population in this region was obese or overweight (57.7% in San Diego County and 69.8% in Imperial County)

Diabetes:

- The diabetes prevalence along the U.S./Mexico border region is 2-3 times higher than that of the United States
- In 2009, Imperial County reported an adult diabetes prevalence that was one of the highest in California (9.6%)
- In San Diego County, Hispanic adults (10.5%) have a higher prevalence of diabetes compared to non-Hispanic White adults (4.9%) and all races combined (7.8%)

Access to Care

- In San Diego County 90.9% of the population has health insurance (94.5% of Whites but only 82.1% of Hispanics)
- In Imperial County 86.9% of the population has health insurance (87.4% of Whites and 86.0% of Hispanics)

Environmental Health

- San Diego and Imperial County share air basins with major metropolitan areas in Mexico
- These air basins are considered to be in nonattainment of EPA standards for ozone and particulate pollution
- Asthma related hospitalizations for children are significantly higher in Imperial County compared to the rest of California

California Department of Public Health: http://www.cdph.ca.gov/Pages/DEFAULT.aspx
California Department of Social services: http://www.cdss.ca.gov/refugeeprogram/PG1537.htm
California Health Interview Survey, 2009: http://www.askchis.com/
Pan-American Health Organization, 2010: http://new.paho.org/sur/index2.php?option=com_content&do_pdf=1&id=238
RITA: http://www.bts.gov/programs/international/transborder/TBDR_BC/TBDR_BCQ.html
U.S. Census Bureau: http://www.census.gov/
U.S. Department of Agriculture: http://www.agcensus.usda.gov/Publications/2007/Full_Report/Volume_1,_Chapter_2_US_State_Level/
U.S. Department of Indian Affairs: http://www.bia.gov/WhoWeAre/RegionalOffices/Western/index.htm
University of San Diego: http://www.sandiego.edu/nativeamerican/reservations.html

Additional information on California Border Health issues can be found at: http://ww2.cdph.ca.gov/programs/cobbh

Appendix 2. California Border Health Collaborative Strategic Plan

CALIFORNIA BORDER HEALTH COLLABORATIVE
STRATEGIC PLAN

OUR VISION IS: **United for a healthy border region**

OUR MISSION IS: To enhance communication, collaboration and coordination among public, private and academic institutions in order to protect and improve the health of individuals, families and communities along the California/Baja California border region.

OUR VALUES ARE: ***Commitment*** ***Inclusivity*** ***Equity*** ***Sustainability***

LONG-TERM GOALS, STRATEGIES AND OBJECTIVES:
What we will do, how we will do it, and the actions we will take

COMMUNICATE

What: Communicate with stakeholders, funders and policy makers to address emerging public health needs, gaps and disparities affecting individuals, families and communities.

- ***How**: Adopt a proactive approach to communicate a unified agenda for advocacy, education and funding priorities.*
- **Actions:**
  - Launch health education and awareness initiatives aimed at target audiences utilizing electronic and social media
  - Create a clearinghouse of information on health issues and resources specific to border regions

COLLABORATE

What: Collaborate on approaches, policies, and practices to address shared priorities in health.

- ***How:** Seek new partners to strengthen the impact of the Collaborative.*
- **Actions:**
  - Increase membership in the Collaborative
  - Create inventory of existing organizational initiatives that could be broadened or enhanced through collaboration
- ***How:** Provide opportunities for partners to identify shared goals, policies and initiatives.*
- **Actions:**
  - Establish workgroups around key focus areas or initiatives
  - Provide a venue for organizations, policymakers and donors to come together to exchange information on border health issues
  - Expand capacity for advocacy

COORDINATE

What: Coordinate efforts by leveraging resources and aligning activities to ensure sustainability and improve health outcomes.

- ***How:** Devise an infrastructure that allows for the successful operation of the Collaborative*
- **Actions:**
  - Conduct asset and needs mapping to help identify critical areas and opportunities to inform Collaborative work
  - Identify potential funding sources or opportunities, inside and outside of the Collaborative, that align with the Collaborative's goals
- ***How:** Maximize the resources and activities of the partners to advance the goals of the Collaborative*
- **Actions:**
  - Secure and/or leverage additional resources to support joint activities on border health
  - Conduct joint activities or initiatives that produce measurable impacts on border health
